# RETRACTION: The Impact of Inflammatory Biomarkers on Amputation Rates in Patients with Diabetic Foot Ulcers

**DOI:** 10.1111/iwj.70164

**Published:** 2024-12-15

## Abstract

**RETRACTION:** XuY. , GengR. , MengX. , FengZ. , WangX. , ZhangG. , and BaiL. , “The Impact of Inflammatory Biomarkers on Amputation Rates in Patients with Diabetic Foot Ulcers,” International Wound Journal 21, no. 4 (2024): e14827, 10.1111/iwj.14827.38522433 PMC10961172

The above article, published online on 24 March 2024, in Wiley Online Library (http://onlinelibrary.wiley.com/), has been retracted by agreement between the journal Editor in Chief, Professor Keith Harding; and John Wiley & Sons Ltd. Following an investigation by the publisher, all parties have concluded that this article was accepted solely on the basis of a compromised peer review process. The editors have therefore decided to retract the article. The authors responded to our notice regarding the retraction but did not state their agreement nor their disagreement.

**RETRACTION:** Y. Xu , R. Geng , X. Meng , Z. Feng , X. Wang , G. Zhang , and L. Bai , “The Impact of Inflammatory Biomarkers on Amputation Rates in Patients with Diabetic Foot Ulcers,” International Wound Journal 21, no. 4 (2024): e14827, 10.1111/iwj.14827.38522433 PMC10961172

The above article, published online on 24 March 2024, in Wiley Online Library (http://onlinelibrary.wiley.com/), has been retracted by agreement between the journal Editor in Chief, Professor Keith Harding; and John Wiley & Sons Ltd. Following an investigation by the publisher, all parties have concluded that this article was accepted solely on the basis of a compromised peer review process. The editors have therefore decided to retract the article. The authors responded to our notice regarding the retraction but did not state their agreement nor their disagreement.

